# Supplementary material for: ProteinShader: illustrative rendering of macromolecules
Source: BMC Struct Biol. 2009 Mar 30;9:19. doi: 10.1186/1472-6807-9-19 (PMC2672931; doi:10.1186/1472-6807-9-19)
Supplement: Additional file 1 — ProteinShader program without source code. This compressed file contains the complete ProteinShader program including associated libraries, but no source code. A README.txt file gives an overview of the ProteinShader distribution, and the index.html file in the help subdirectory has directions on getting started with the program as well as a set of tutorials. [file 1472-6807-9-19-S1.zip › ProteinShader-beta-0_9_4-binary/help/api/org/proteinshader/graphics/enums/class-use/ShaderEnum.html]

Uses of Class org.proteinshader.graphics.enums.ShaderEnum (ProteinShader API)


|  |  |  |
| --- | --- | --- |
| **Overview** \| **Package** \| **Class** \| **Use** \| **Tree** \| **Deprecated** \| **Index** \| **Help** | |  |
| PREV   NEXT | **FRAMES**    **NO FRAMES**     **All Classes** |  |


---


## **Uses of Class org.proteinshader.graphics.enums.ShaderEnum**

| Packages that use ShaderEnum | |
| --- | --- |
| **org.proteinshader.graphics.enums** | Holds the enums needed by org.proteinshader.graphics and its subpackages. |
| **org.proteinshader.graphics.shader** | Holds the classes needed for compiling and managing the vertex and fragment shaders used for texture mapping and custom lighting calculations. |

Uses of ShaderEnum in org.proteinshader.graphics.enums

| Methods in org.proteinshader.graphics.enums that return ShaderEnum | |
| --- | --- |
| `static ShaderEnum` | `ShaderEnum.valueOf(String name)`             Returns the enum constant of this type with the specified name. |
| `static ShaderEnum[]` | `ShaderEnum.values()`             Returns an array containing the constants of this enum type, in the order they're declared. |

Uses of ShaderEnum in org.proteinshader.graphics.shader

| Methods in org.proteinshader.graphics.shader that return ShaderEnum | |
| --- | --- |
| `ShaderEnum` | `ShaderProgram.getPair()`             Returns the type of shader pair (vertex and fragment pair). |

| Methods in org.proteinshader.graphics.shader with parameters of type ShaderEnum | |
| --- | --- |
| `ShaderProgram` | `ShaderProgramFactory.compile(GL gl, ShaderEnum pair)`             Reads a pair of vertex and fragment shader files and then compiles and links them. |
| `ShaderProgram` | `ShaderProgramFactory.createShaderProgram(GL gl, File vertFile, File fragFile, ShaderEnum pair)`             Reads source code files for a vertex shader and a fragment shader and creates an OpenGL shader program object. |
| `ShaderProgram` | `ShaderProgramFactory.createShaderProgram(GL gl, String vertFilename, String fragFilename, ShaderEnum pair)`             Reads source code files for a vertex shader and a fragment shader and creates an OpenGL shader program object. |
| `void` | `ShaderProgram.setPair(ShaderEnum pair)`             Sets the type of shader pair (vertex and fragment pair). |

Constructors in org.proteinshader.graphics.shader with parameters of type ShaderEnum

`ShaderProgram(File vertFile,
File fragFile,
ShaderEnum pair)`
  
          Constructs a ShaderProgram object to store info on an OpenGL
Shading Language "program" object.

---


|  |  |  |
| --- | --- | --- |
| **Overview** \| **Package** \| **Class** \| **Use** \| **Tree** \| **Deprecated** \| **Index** \| **Help** | |  |
| PREV   NEXT | **FRAMES**    **NO FRAMES**     **All Classes** |  |


---

# *Copyright © 2007-2008*
